# Effects of green light-emitting diode irradiation on hepatic differentiation of hepatocyte-like cells generated from human adipose-derived mesenchymal cells

**DOI:** 10.1038/s41598-023-45967-7

**Published:** 2023-11-15

**Authors:** Yuhei Waki, Yu Saito, Shuhai Chen, Tetsuya Ikemoto, Takayuki Noma, Hiroki Teraoku, Shinichiro Yamada, Yuji Morine, Mitsuo Shimada

**Affiliations:** Department of Surgery, Tokushima University, 3-18-15 Kuramoto-cho, Tokushima, 770-8503 Japan

**Keywords:** Mesenchymal stem cells, Stem-cell differentiation, Lasers, LEDs and light sources

## Abstract

Light-emitting diode (LED) irradiation has been used in the differentiation of mesenchymal stem cells into a variety of cell types. This study investigated the effect of green LED (GLED) irradiation on the differentiation of adipocyte-derived mesenchymal cells into hepatocyte-like cells (HLCs) and the mechanism of its action. HLCs in the hepatocyte maturation phase were irradiated with GLED (520 nm, 21 W/m^2^, 5 min/day for 10 days). The cells were then assessed for expression of hepatocyte maturity genes and opsin 3 (OPN3), hepatocyte function, viability, apoptosis, and levels of reactive oxygen species (ROS), intracellular adenosine triphosphate (ATP) and calcium ions (Ca^2+^). GLED irradiation increased Alpha-1 antitrypsin and Ornithine transcarbamylase gene expression, promoted Cytochrome P450 3A4 activity and urea synthesis, and elevated intracellular ROS, ATP and Ca^2+^ levels. OPN3 expression was significantly more upregulated in GLED-irradiated HLCs than in the non-irradiated HLCs. No significant difference in cell viability or apoptosis was observed between GLED-irradiated and non-irradiated HLCs. GLED irradiation can promote hepatocyte maturation and functions through OPN3. GLED irradiation also stimulated mitochondrial function via Ca^2+^/ATP/ROS activation. GLED irradiation has potential to support cell-based transplantation in patients.

## Introduction

Hepatocyte transplantation has been used as a bridging therapy for patients with metabolic diseases and acute liver failure, mainly in Europe and the United States. However, there are various problems with hepatocyte transplantation, including a shortage of donors, poor cell survival rates following isolation and cryopreservation, and poor long-term cell survival rates after transplantation. To address these donor source problems, the differentiation of hepatocytes from induced pluripotent stem cells, embryonic stem cells, and mesenchymal stem cells (MSCs) has been actively pursued.

We have previously reported methods for inducing differentiation of human adipose tissue-derived mesenchymal stem cells (ADSCs) into hepatocyte-like cells (HLCs), which acquire hepatocyte functions, such as ammonia clearance and CYP3A4 enzyme activity^1^. However, our HLCs have inferior hepatocyte function compared with primary hepatocytes, indicating that improved hepatocyte function is necessary for these cells to be used clinically.

Recently, light-emitting diode (LED) irradiation has been used to treat several clinical conditions, including wound healing, pain, bone regeneration and treatment of various diseases and infections^2^. We have reported that LED irradiation can result in various biological effects, such as mouse hepatocyte proliferation and colon cancer cell apoptosis^3,4^. The effect of LED irradiation at different wavelengths and energy densities also improves the regenerative capacity of various types of MSC^5,6^. Green LED (GLED) irradiation promotes the differentiation of MSCs into neurons and osteoblasts^2,7^. However, it is not known whether GLED can promote the hepatic differentiation of HLCs. Therefore, we focused on the effect of GLED on the differentiation of HLCs from ADSCs.

Opsins are G protein-coupled receptors (GPCRs) present in most animals that absorb light to activate visual and non-visual functions^8^. Among seven opsin subtypes, opsin 3 (OPN3) is expressed in various human tissues, including the liver, brain and kidney, in addition to the eye^9,10^. OPN3 can induce activation of calmodulin-dependent protein kinase (CaMK) and p38 through a GPCR-related phosphorylation pathway^11^. In addition, CaMK can activate peroxisome proliferator-activated receptor gamma coactivator 1-alpha (PGC-1A), which is a master gene related to mitochondrial metabolism, to cause increased expression of genes involved in urea synthesis, including ornithine transcarbamylase (OTC)^12^. However, the effects and mechanisms of GLED irradiation on hepatic differentiation of HLCs generated from ADSCs is unclear. In the present study, therefore, the effects of GLED irradiation on the differentiation of HLCs from ADSCs was investigated, and its mechanism was also explored.

## Results

### Morphology and cell viability

**Figure 1:**
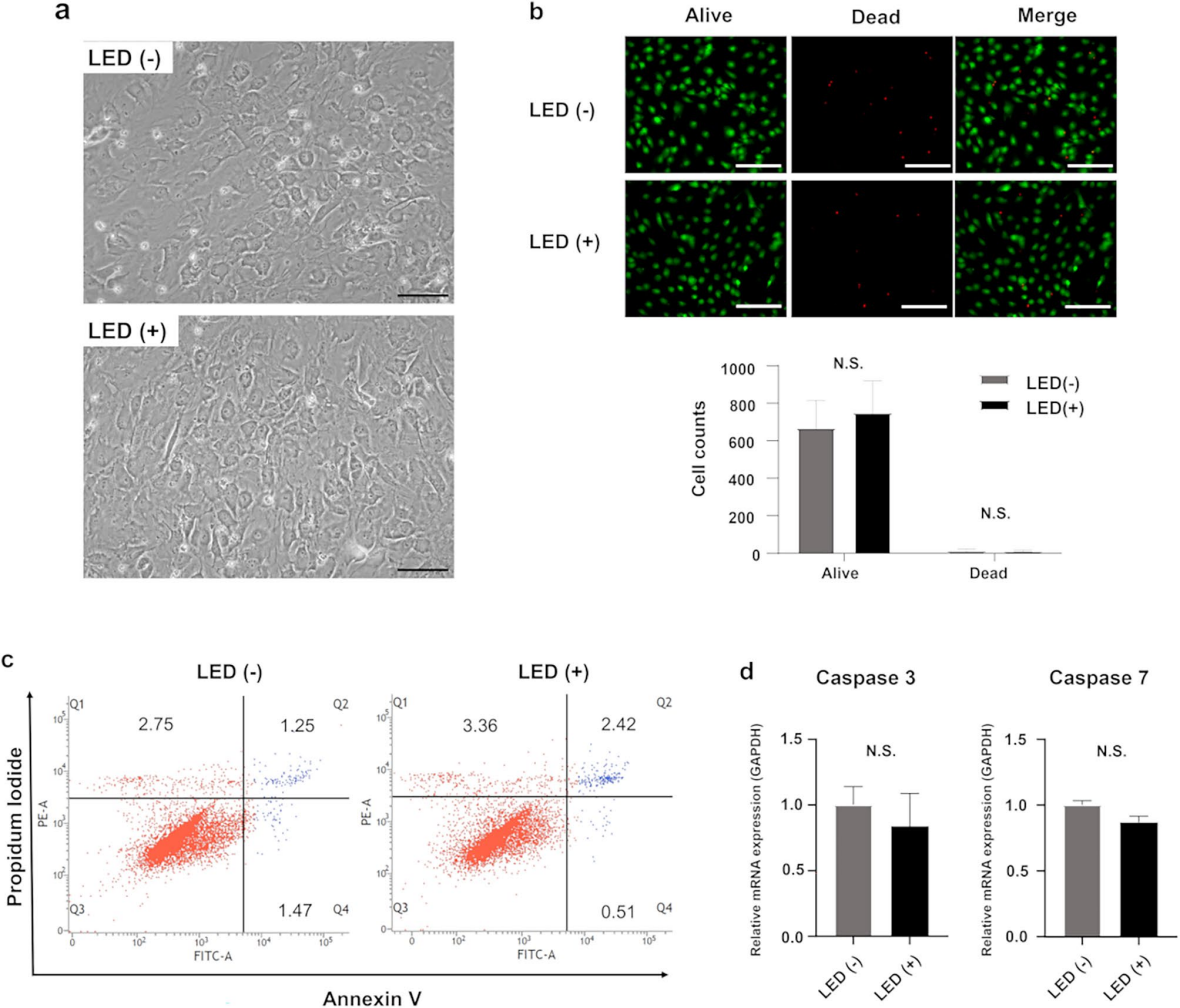
Morphology, viability and apoptosis of HLCs with or without GLED irradiation on day 21. (**a**) The morphology of non-irradiated (LED−) and GLED-irradiated (LED+) HLCs. Scale bar = 100 µm. (**b**) Viability of HLCs irradiated by GLED was assessed by live/dead staining, and no significant difference in viable cell counts between GLED-irradiated and non-irradiated HLCs was observed. Scale bar = 200 µm. (**c**) Annexin/propidium iodide flow cytometry demonstrated no significant difference between GLED-irradiated and non-irradiated HLCs. (**d**) Expression of caspase 3 and 7 genes was not significantly different between GLED-irradiated and non-irradiated HLCs (n = 4). The data are shown as means ± standard deviation. N.S., not significant.

There were no significant differences in morphology between GLED-irradiated and non-irradiated cells, with both showing morphology similar to our HLCs previously reported^1^ (Fig. 1a). As shown in Fig. 1b, live/dead images demonstrated no significant difference between GLED-irradiated and non-irradiated HLCs. Annexin/PI flow cytometry and caspase 3 and 7 gene expression were also not significantly different between the two groups (Fig. 1c and d).

### Hepatocyte genes and functions

**Figure 2:**
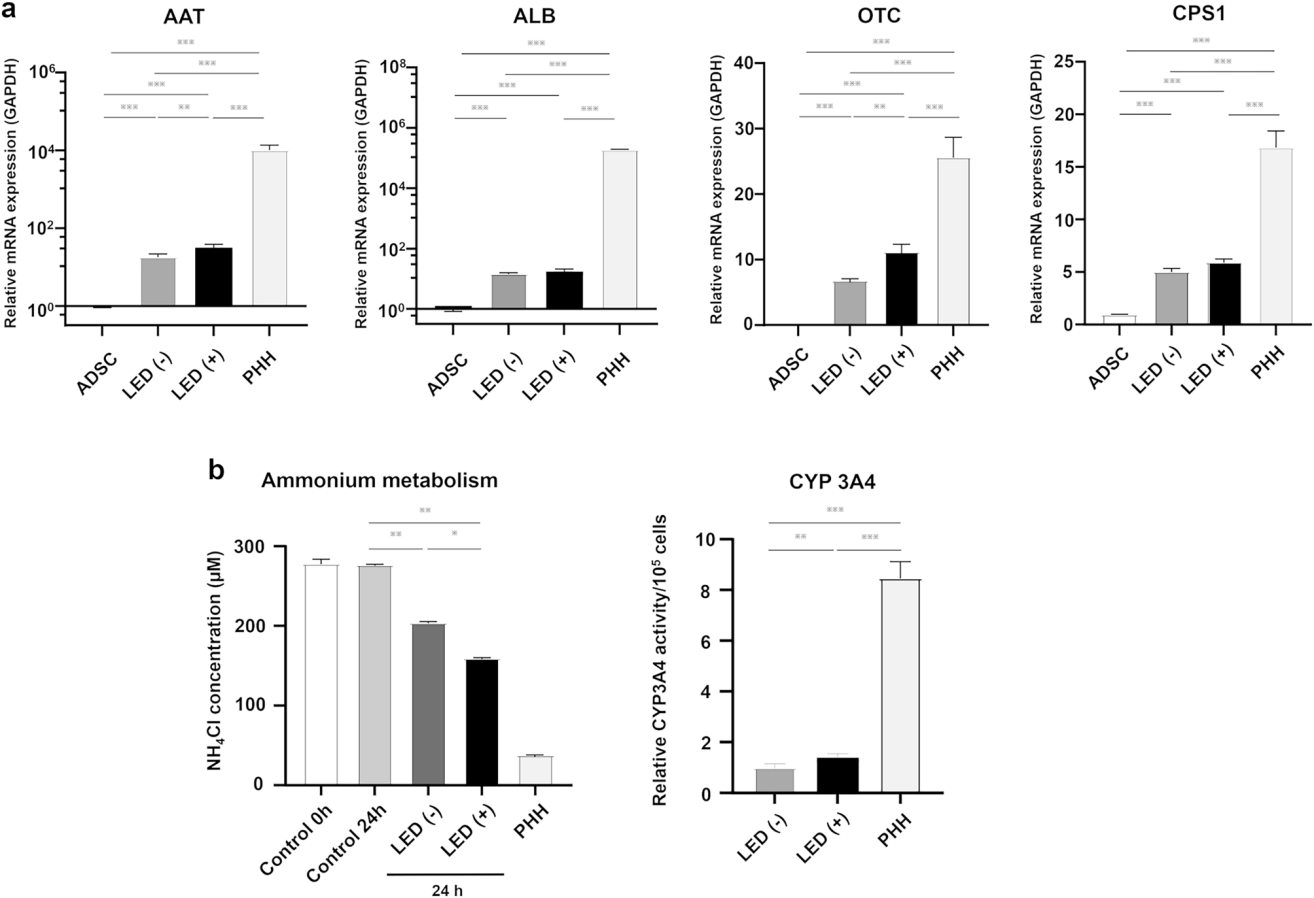
Hepatocyte maturation genes and hepatocyte function in GLED-irradiated and non-irradiated HLCs at day 21. (**a**) RT-PCR showed that *AAT* and *OTC* expression was significantly higher in GLED-irradiated HLCs compared with non-irradiated HLCs (n = 4). PHHs were used as a positive control (n = 4). (**b**) Ammonia assays showed significantly higher resolution in GLED-irradiated HLCs than in non-irradiated HLCs, and CYP3A4 activity was significantly higher in GLED-irradiated HLCs than in non-irradiated HLCs, but these hepatocyte functions did not reach to PHHs. The data are shown as means ± standard deviation. * P < 0.05, ** P < 0.01 and *** P < 0.001. ADSC, adipose tissue-derived mesenchymal stem cell; AAT, alpha-1 antitrypsin transcarbamylase; ALB, albumin; OTC, ornithine transcarbamylase; CPS1, carbamyl phosphate synthase 1, PHH, primary human hepatocyte.

The levels of *AAT* and *OTC* mRNAs were significantly higher in GLED-irradiated HLCs compared with non-irradiated HLCs but there were no differences in the mRNA levels of *ALB* and *CPS1* between the two groups (Fig. 2a). All the levels of hepatocyte genes of GLED-irradiated HLCs were significantly lower compared to primary human hepatocytes (PHHs) (Fig. 2a). Consistently, GLED irradiation improved both ammonium clearance and CYP3A4 activity in HLCs, but did not reach to PHHs (Fig. 2b). There was no difference between the GLED-irradiated and non-irradiated HLCs in the concentration of albumin in the culture medium on Day 21 (Supplemental Fig. 1).

### OPN3 expression

**Figure 3:**
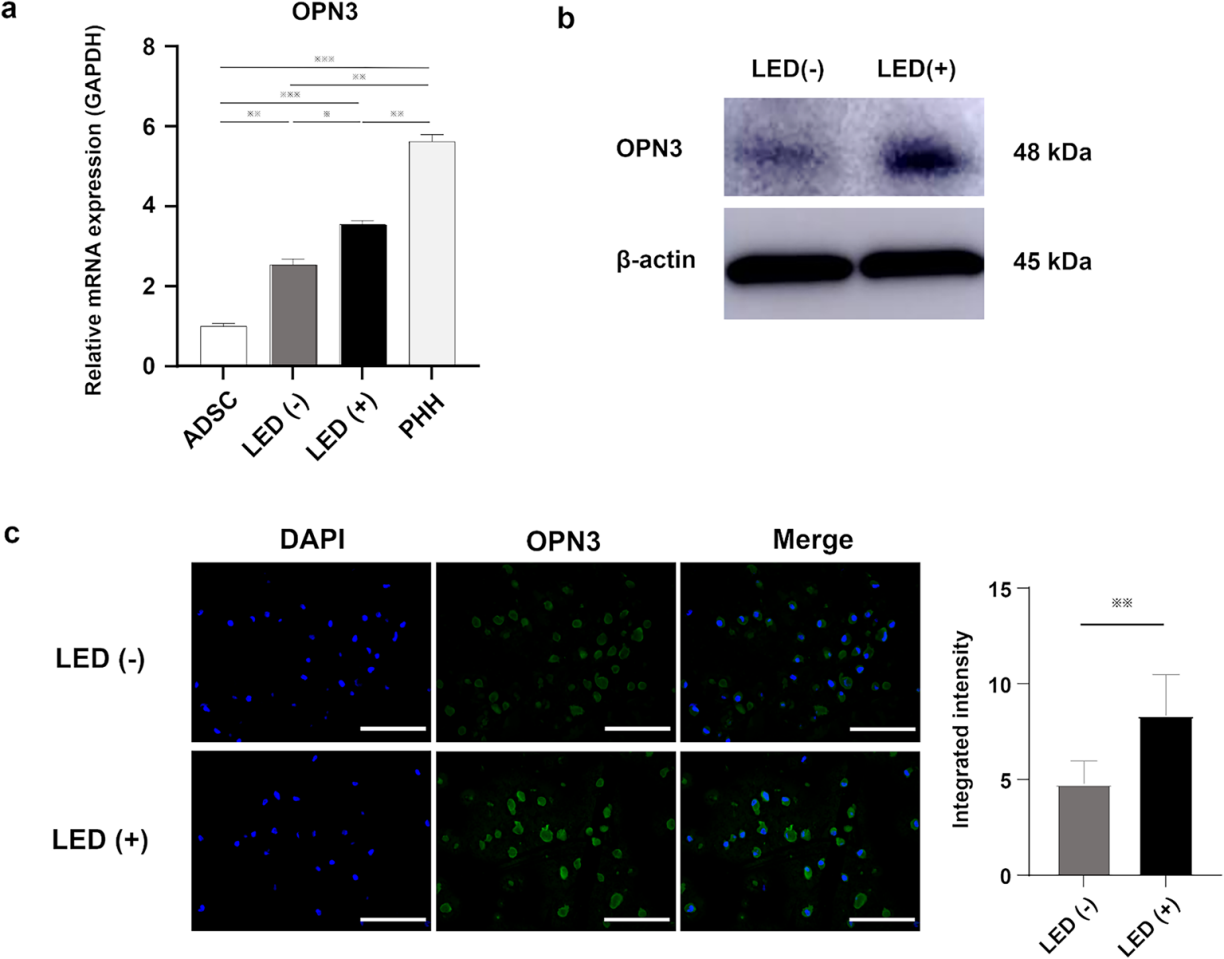
Expression of OPN3 in GLED-irradiated HLCs. (**a**) Gene expression of *OPN3* was significantly higher in GLED-irradiated HLCs than in non-irradiated HLCs (n = 4). (**b**) Western blotting demonstrated that OPN3 protein expression was more abundant in GLED-irradiated HLCs than in non-irradiated HLCs. (**c**) Fluorescence immunostaining showed higher OPN3 intensity in GLED-irradiated HLCs than in non-irradiated HLCs. The data are shown as means ± standard deviation. * P < 0.05 and ** P < 0.01. OPN3, opsin 3, ADSC, adipose-derived stem cell, PHH, primary human hepatocyte.

OPN3 expression was evaluated by RT-PCR (Fig. 3a), western blot analysis (Fig. 3b and Supplementary Fig. 2) and immunofluorescence staining (Fig. 3c). Each analysis showed OPN3 to be upregulated in GLED-irradiated HLCs compared with levels in non-irradiated HLCs. Meanwhile, the level of OPN3 expression in GLED-irradiated HLCs was significantly lower compared to PHHs.

### Mitochondrial function

**Figure 4:**
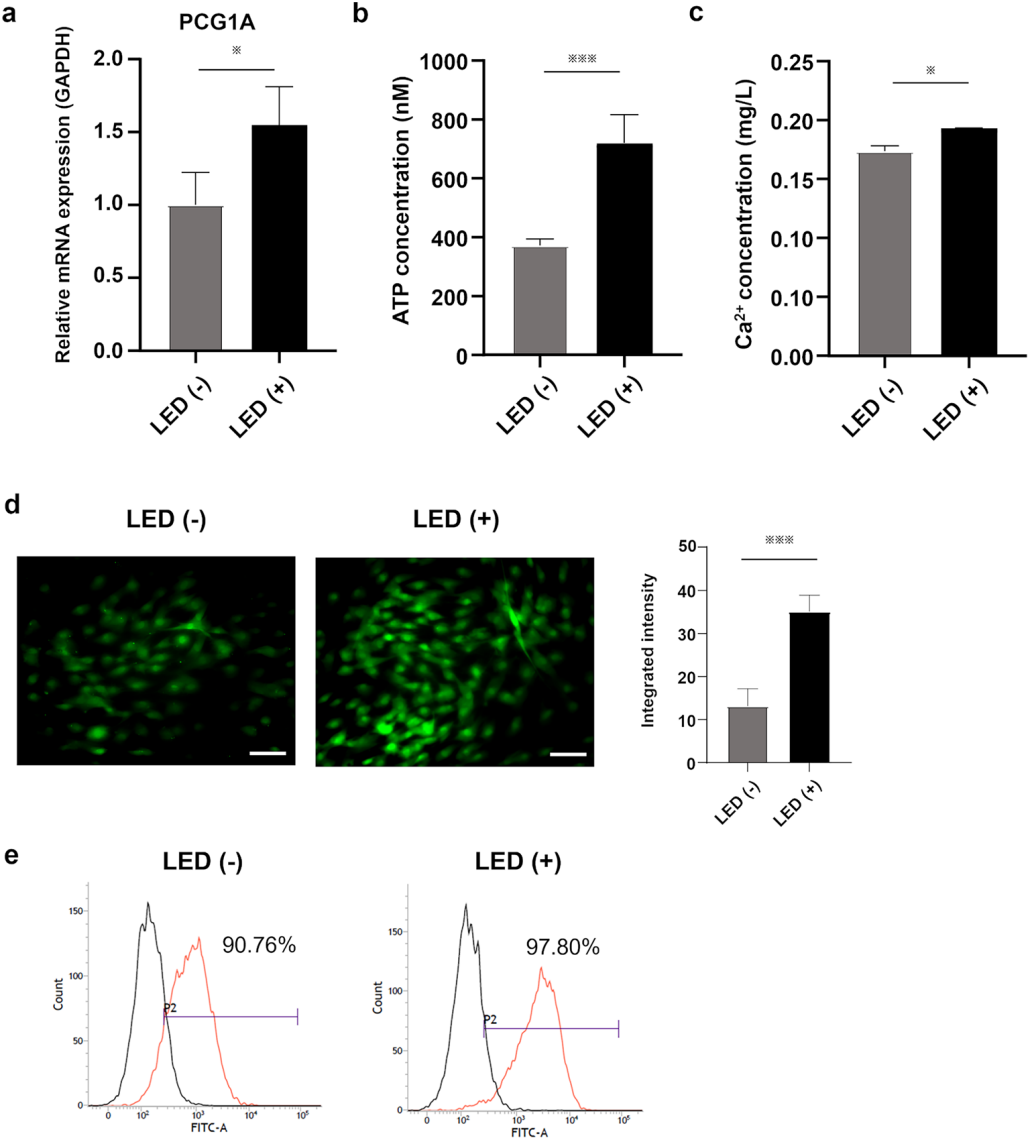
Mitochondrial function in GLED-irradiated HLCs on day 21. (**a**) RT-PCR showed that *PCG1A* expression was significantly higher in GLED-irradiated HLCs compared with non-irradiated HLCs (n = 4). (**b, c**) GLED-irradiated HLCs exhibited higher levels of intracellular Ca^2+^ and ATP compared with non-irradiated HLCs (n = 4). (**d**) ROS activity, fluorescently stained with DCFDA, was more intense in GLED-irradiated HLCs than in non-irradiated HLCs. (**e**) Flow cytometry analysis showed a higher percentage of cells with ROS activity for GLED-irradiated HLCs compared with non-irradiated HLCs. The data are shown as means ± standard deviation. * P < 0.05 and *** P < 0.001. PGC-1A, peroxisome proliferator-activated receptor gamma coactivator 1-alpha; ATP, adenosine triphosphate; ROS, reactive oxygen species; DCFDA, 2′,7′-dichlorodihydrofluorescin diacetate.

Compared with non-irradiated cells, the mRNA level of PCG1A, which is a master gene of mitochondrial metabolism, was significantly increased (Fig. 4a) and intracellular ATP production and calcium ions (Ca^2+^) concentration were higher in GLED-irradiated HLCs (Fig. 4b and c). DCFDA staining was more intense in GLED-irradiated HLCs and flow cytometry assessment showed higher ROS activity in GLED-irradiated HLCs compared with non-irradiated cells (Fig. 4d and e).

## Discussion

The present study revealed that GLED irradiation of HLCs elevated the expression of hepatocyte maturation and urea synthesis genes, and improved hepatocyte functions, such as ammonia clearance and CYP3A4 activity. Moreover, OPN3 was upregulated by GLED irradiation. As a result, PGC1A gene expression and intracellular ATP, Ca^2+^ and ROS levels were all higher in GLED-irradiated HLCs than in non-irradiated HLCs. Therefore, GLED irradiation may improve hepatocyte maturation and function through OPN3 and Ca^2+^/ROS/ATP interactions via PCG1A upregulation during the hepatocyte differentiation process.

HLCs derived from ADSCs are considered to have promise as a bridging therapy for metabolic disorder syndrome or acute liver failure following liver transplantation. ADSCs are considered to be an ideal cell source for hepatocytes because their use has no prohibitive ethical issues, they lack genetic damage and can be isolated from a patient with minimal invasiveness. We previously demonstrated that both 2D- and 3D-cultured HLCs can be generated from human ADSCs in 21 days^1^. Of these HLCs, 3D-HLCs had comparable hepatocyte function to other reported HLCs, showing similar, but not equivalent, hepatocyte function to primary hepatocytes. Therefore, the differentiation process from ADSCs to HLCs needs improvement^13^.

LED irradiation has positive effects on stimulation of neuronal growth, protection of post-ischemia skeletal muscle, protection of cardiomyocytes, acceleration of wound healing, reduction of the inflammatory response, and stimulation of cell proliferation and differentiation^3,5,7,14^. Here, we demonstrated that GLED irradiation also has an effect on the function of HLCs. GLED irradiation promoted the expression of hepatocyte maturation genes and hepatocyte functions, such as CYP3A4 activity and ammonia reduction, which might bring HLC function a little closer to those of primary human hepatocytes. A previous study showed that a GLED device working in an incubator enhanced the therapeutic efficacy of human ADSCs by modulating photoreceptor expression to promote advanced wound healing^15^. A GLED irradiation device may, therefore, be used in an incubator to improve the hepatocyte function of HLCs.

LEDs are superior to other light resources in their functions of controlling light intensity and wavelength^4^. Previous study has reported that GLED strongly accelerated neural differentiation of human umbilical cord-derived mesenchymal cells, compared to red LED^14^. In addition, the optimal energy densities for cell differentiation have reported to range from 0.1 to 6 J/cm^2^^16^. This study demonstrated that GLED irradiation (520 nm, 0.63 J/cm^2^, ten times) promoted hepatic differentiation of HLCs. Meanwhile, the hepatic maturation gene expression of HLCs with GLED irradiation for 10 min (1.26 J/cm^2^) for ten days was as same as that with non-irradiation (Supplemental Fig. 3). Therefore, 5 min’ GLED irradiation in the hepatocyte differentiation phase could be effective for hepatic maturation.

GLEDs exert their effects by activating opsins, which are GPCRs associated with several phosphorylation pathways^8^. Increased OPN3 expression by GLED irradiation promotes kinase activation and increased ATP production in human orbital adipose stem cells, and phototransduction through OPN3 regulates the selectivity of phosphorylation by target kinases^17^. Additionally, OPN3 can induce CaMK and p38 through a GPCR pathway, and can ultimately upregulate matrix metalloproteinase in human dermal fibroblasts by activating Ca^2+^^11^. Activation of CaMK and p38 enhances PCG1A expression, which promotes the expression of OTC and CPS1. These genes are involved in urea synthesis and the differentiation of human stem cells into astrocytes^12,18,19^.

OPN3 significantly increases intracellular ATP or Ca^2+^, which are parameters of mitochondrial function, through activation of transient receptor potential channels, resulting in the generation of ROS^20,21^. Intracellular Ca^2+^ elevation can cause ROS generation^22^; therefore, an increase in intracellular ROS levels may trigger the activation of OTC gene transcription in hepatocyte differentiation^16^. Importantly, our results of GLED irradiation leading to a significant increase in intracellular ATP and Ca^2+^ levels in HLCs are in line with the above findings. Therefore, in addition to the OPN3 pathway described above, ROS may be involved in the upregulation of OTC gene expression.

**Figure 5:**
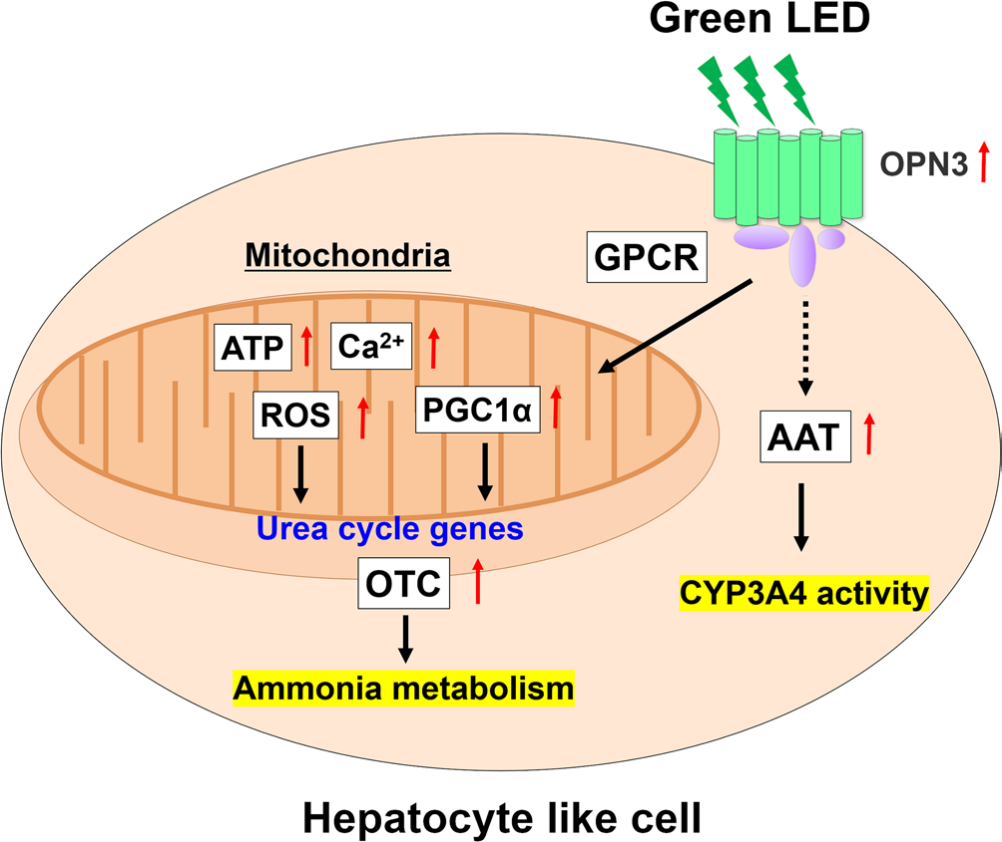
Schematic representation of proposed mechanisms underlying improved hepatocyte maturation and function in response to GLED irradiation of HLCs. OPN3, opsin 3; GPCR, G protein-coupled receptor; AAT, alpha-1 antitrypsin transcarbamylase; OTC, ornithine transcarbamylase; ATP, adenosine triphosphate; ROS, reactive oxygen species; peroxisome proliferator-activated receptor gamma coactivator 1-alpha, PGC-1A.

We suggest that GLED irradiation increases CYP3A4 activity by activation of the OPN3 GPCR pathway. These findings provide new insights into the molecular mechanisms underlying the action of hepatic maturation genes. Figure 5 summarizes both the effect of GLED irradiation and its mechanism of action.

There are certain limitations to the present study. The results were based on in vitro experiments. Currently, we are planning to transplant our HLCs (non-irradiated) into mouse models of hepatic metabolic disorders. Further investigation is warranted in in vivo animal models of hepatic metabolic disorders to evaluate the effects of LED-irradiated HLCs. In conclusion, GLED irradiation can promote hepatocyte maturation and functions through OPN3. GLED irradiation stimulated mitochondrial function via Ca^2+^/ATP/ROS activation. GLED irradiation might be a potential method to support cell-based transplantation in patients.

## Methods

### Cell culturing

PHHs were purchased from Thermo Scientific Inc. (Waltham, MA, USA), and cultured using the Hepatocyte Medium (Thermo Scientific Inc.) according to the manufacturer’s protocol. PHHs were used for experiments within 3 days after seeding.

### HLC generation

STEMPRO human ADSCs were purchased from Life Technologies (Tokyo, Japan). Our HLC generation protocol has been reported^1^. ADSCs were used in experiments at passages 2–6. Briefly, ADSCs (2 × 10^6^ per well) were seeded in 6-well flat-bottomed collagen-coated plates (Nunclon Sphera Microplates, Thermo Scientific Inc.) and incubated with serum-free Dulbecco’s modified Eagle’s medium with F-12 Supplement (DMEM/F-12) for 24 h. Thereafter, our three-step differentiation protocol was performed. In the first step, to induce definitive endoderm differentiation, cells were incubated with DMEM/F-12 containing 0.5 mg/m bovine serum albumin fraction V (BSA; Sigma- Aldrich, St. Louis, MO, USA) and 2 µmol/L Chir99021 (a glycogen synthase kinase 3 inhibitor; Selleckchem, Tokyo, Japan) for 24 h. The following day, 1% insulin-transferrin-selenium (ITS, Sigma-Aldrich) was added to the medium. In the second step, for hepatoblast differentiation, the medium was changed to minimum essential medium with non-essential amino acids (Thermo Scientific Inc.) containing 0.5 mg/mL BSA, 1% ITS, 20 ng/mL bone morphogenic protein 2 (PeproTech, Inc. NJ, USA), and 30 ng/mL fibroblast growth factor 4 (PeproTech, Inc.) and incubation continued for 5 days. In the third step, for induction of hepatocyte differentiation, the cells were treated with 20 ng/mL hepatocyte growth factor (PeproTech, Inc.) for 5 days, followed by treatment with 20 ng/mL hepatocyte growth factor, 10 ng/mL oncostatin M (PeproTech, Inc.) and 1 × 10^−6^ M dexamethasone (Sigma-Aldrich) for another 5 days.

### GLED irradiation

**Figure 6:**
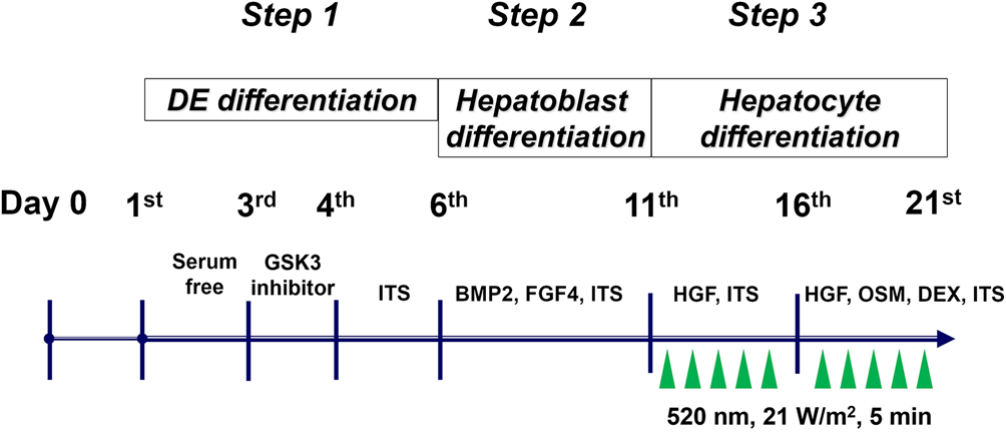
Study protocol of GLED-irradiated HLCs. GLEDs were irradiated once a day for 5 min at 21 W/m^2^ during the 10 days of the hepatocyte differentiation phase. DE, definitive endoderm; GSK, glycogen synthase kinase; ITS, insulin-transferrin-selenium; BMP, bone morphogenic protein; FGF, fibroblast growth factor; HGF, hepatocyte growth factor; OSM, oncostatin M; DEX, dexamethasone.

An LED irradiation device (3LH-256/3LH-75DPS, Nippon Medical & Chemical Instruments Co., Ltd., Osaka Japan) was used to produce GLED with a wavelength of 520 nm at maximum light intensity. A photoradiometer (Light analyzer LA-105, Nippon Medical & Chemical Instruments Co., Ltd.) was used to measure the light intensity. The cell culture plates were placed on the LED irradiation device and energy density generation was set at 21 W/m^2^. Cells were exposed to GLED in the third step (hepatocyte differentiation phase) at room temperature for 5 min which gave a radiant exposure of 0.63 J/cm^2^ for each day. The GLED irradiation protocol is shown in Fig. 6. Control group cells were treated in the same manner except for no GLED exposure. Sufficient ventilation was provided to ensure that the temperature of the culture medium did not change during treatment. The experiments were replicated at least 3 times under the same conditions with three purchased ADSCs.

### Cell viability

The LIVE/DEAD Cell Imaging Kit (Thermo Fisher Scientific, Inc.) was used to determine the live/dead nucleated cells among GLED-treated and non-treated HLCs at day 21, following the manufacturer’s protocol. The fluorescence signals were measured using a fluorescence microscope (Keyence Corporation). Cells were counted using Image J software (ver. 1.53, National Institutes of Health, Bethesda, MD, USA).

### Apoptosis detection by flow cytometry

Flow cytometry was used to detect apoptosis. Both irradiated and non-irradiated cells were labeled with Annexin V-conjugated fluorescein isothiocyanate and propidium iodide (PI) using a MEBCYTO Apoptosis Kit according to the manufacturer’s instructions (MBL international, Woburn, MA, USA). Quantitative analysis was performed using a FACSVerse cytometer and FACSiute software (Becton Dickinson, Franklin Lakes, NJ, USA).

### RT-PCR of HLCs

Total RNA was prepared from each HLC sample using the RNeasy Mini Kit (Qiagen, Hilden, Germany) according to the manufacturer’s instructions. cDNA was synthesized using a reverse transcription kit (Applied Biosystems, Foster City, CA, USA). The following TaqMan assays (with assay identification number and primers) were used: alpha-1 antitrypsin (AAT) (Hs00165475_m1), albumin (ALB) (Hs00910225_m1), OTC (Hs00166892_m1), carbamyl phosphate synthase 1 (CPS1) (Hs00157048_m1), OPN3 (Hs00173892_m1) and PGC-1A (Hs00173304_m1). GAPDH (Hs02786624_g1) was used as an internal control with stable level of expression for normalization (Supplemental Table 1). All primers were purchased from Thermo Fisher Scientific, Inc. The data were analyzed using the 2^−ΔΔCt^ method. Relative expression levels were calculated as ratios to *GAPDH* expression. The results are presented as the fold change of the relative mRNA expression for a group compared with that in the control group.

### Western blotting

RIPA buffer (Thermo Fisher Scientific, Inc.) containing a protease inhibitor cocktail (Sigma- Aldrich) and a PhosSTOP phosphatase inhibitor cocktail (Roche, Tokyo, Japan) was used for protein extraction. Total protein concentrations were assessed with a BCA Kit (Thermo Fisher Scientific, Inc.) and equal quantities of extracted proteins were separated on 10% sodium dodecyl sulfate–polyacrylamide gel electrophoresis gels and transferred to polyvinylidene difluoride membranes (Bio-Rad, Hercules, CA, USA). To evaluate protein abundance, blots were blocked with 5% skimmed milk for 1 h at room temperature and incubated overnight at 4 °C with the following primary antibodies: anti‑OPN3 (1:1,000; SAB2700986; Sigma-Aldrich) and anti‑β‑actin (1:1,000; cat. no. 4970; Cell Signaling Technology, Inc. MA, USA). Blots were then incubated with anti‑rabbit IgG, HRP‑linked (1:2,000; cat. no. 7074; Cell Signaling Technology, Inc.) as the secondary antibody for 1 h at room temperature. The proteins were detected with chemiluminescence (GE, Little Chalfont, Buckinghamshire, UK).

### CYP3A4 activity assay

CYP3A4 enzyme activity was assessed using the P450- Glo™ CYP3A4 Assay with Luciferin-IPA (Promega Corporation. WI, USA) according to the manufacturer’s instructions. Luminescence was measured using a microplate reader (SpectraMax i3; Molecular Devices, LLC). Luminescent measurements were normalized to the total amount of viable cells.

### Ammonium metabolism assay

Ammonium metabolism was evaluated by the change in ammonium ion concentration in the cell culture supernatant at 24 h after the addition of ammonium chloride (NH_4_Cl). Briefly, NH_4_Cl (FUJIFILM Wako Pure Chemical Corporation) diluted with Hanks’ Balanced Salt Solution (HBSS, FUJIFILM Wako Pure Chemical Corporation) to a standard of 300 µmol/L was added to culture plates after the plates were washed twice with HBSS. The plates were placed adjacent to each other in the same incubator and incubated under the same conditions. The supernatants were then collected and applied to an ammonia assay kit (Cell Biolabs) to measure ammonium concentrations at 24-h intervals after NH_4_Cl addition. Controls were culture plates containing only standard ammonia solution. As a reference value, ammonium metabolism assays were performed using PHHs with Hepatocyte Medium in the same manners described above.

### Immunofluorescence staining

HLCs were immobilized in iPGell (Geno staff, Tokyo, Japan) and fixed with 4% paraformaldehyde according to the manufacturer’s protocol. Frozen jellified cells were then sectioned, mounted on glass slides and incubated with an anti-OPN3 primary antibody (Abcam, ab140901) overnight at 4 °C. Cells were then incubated with a fluorophore-conjugated secondary antibody (Thermo Fisher Scientific Inc., A11008) and then with DAPI (Thermo Fisher Scientific Inc., P306931). Slides were observed under a fluorescence microscope (Keyence Corporation, Itasca, IL, USA).

### ***Ca***^***2***+^***and ATP assays***

Intracellular Ca^2+^ concentration in GLED-treated HLCs was measured using a Ca^2+^ detection assay kit (Abcam, Cambridge, UK). Briefly, after cell homogenization and centrifugation at 20,000 × g for 5 min at 4 °C, supernatants were collected. Samples were mixed with chromogenic reagent and incubated at room temperature for 10 min according to the manufacturer’s protocol. The absorbance values of each sample were then measured at 575 nm with a microplate reader. Measurements for each cell lysate sample were standardized at 1 × 10^6^ HLCs.

To evaluate the amount of ATP in GLED-treated and non-treated HLCs, cell homogenization and centrifugation was performed as for the above Ca^2+^ assay and an adenosine triphosphate (ATP) assay kit (FUJIFILM Wako Pure Chemical Corporation) was used according to the manufacturer’s protocol. Measurements for each cell lysate sample were standardized at 1 × 10^6^ HLCs.

### Detection of reactive oxygen species (ROS)

The ROS Assay Kit (Dojindo Lab., Kumamoto, Japan) was used to measure the formation of intracellular ROS. Briefly, HLCs on differentiation day 21 (10 days after GLED irradiation) were seeded into a 6-well dish and incubated with 2′,7′-dichlorodihydrofluorescin diacetate (DCFDA) for 30 min in the dark at 37 °C. After washing twice with HBSS, the cells were incubated with 100 µmol/L hydrogen peroxide for 30 min according to the manufacture’s protocol. The fluorescence signals were measured and analyzed using a FACSVerse cytometer and FACSiute software (Becton Dickinson). A fluorescence microscope (Keyence Corporation) was used to image cells and the level of cellular fluorescence was measured using the Cell Magic Wand plugin for Image J software.

### Statistical analysis

All data are presented as the mean ± standard difference. Statistical analysis and graph production were conducted using GraphPad Prism v7.0 (GraphPad Software, Inc.) and Image J software. Comparisons between two groups were analyzed using the Mann‑Whitney test. P-values less than 0.05 (two‑sided) were considered statistically significant.

### Supplementary Information

Supplementary Figure 1.

Supplementary Figure 2.

Supplementary Figure 3.

Supplementary Table 1.

## Data Availability

The data that support the findings of this study are available from the corresponding author upon reasonable request.
