# Supplementary material for: Effects of green light-emitting diode irradiation on hepatic differentiation of hepatocyte-like cells generated from human adipose-derived mesenchymal cells
Source: Sci Rep. 2023 Nov 15;13:19954. doi: 10.1038/s41598-023-45967-7 (PMC10651838; doi:10.1038/s41598-023-45967-7)
Supplement: Supplementary file 1 — Supplementary Figure 1. [file 41598_2023_45967_MOESM1_ESM.docx]

***Effects of green light-emitting diode irradiation on hepatic differentiation of hepatocyte-like cells generated from human adipose-derived mesenchymal cells.***

Yuhei Waki MD, Yu Saito MD, PhD, FACS^*^, Shuhai Chen MD, Tetsuya Ikemoto MD, PhD, FACS, Takayuki Noma MD, Hiroki Teraoku MD, PhD, Shinichiro Yamada MD, PhD, FACS, Yuji Morine MD, PhD, FACS, Mitsuo Shimada MD, PhD, FACS

Department of Surgery, Tokushima University, 3-18-15 Kuramoto-cho, Tokushima 770-8503, Japan

**
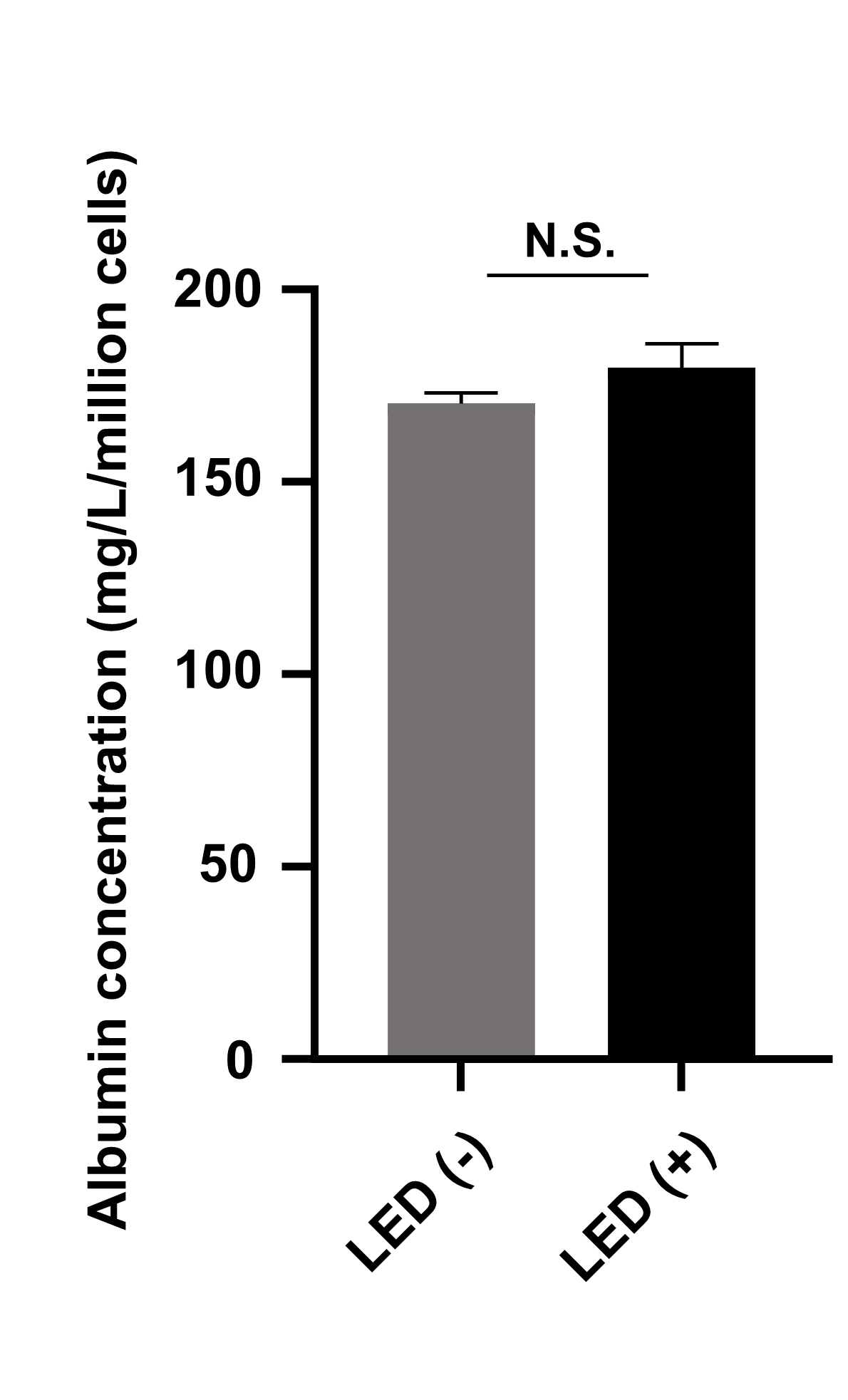
Supplementary Figure 1. Albumin concentration in HLC culture medium on Day 21**

Albumin concentrations in culture medium were comparable between GLED-irradiated HLCs and non-irradiated HLCs. The data are shown as means ± standard deviation. N.S. not significant.

**Material and Method**

Albumin concentration was measured by urine albumin test (SRL, Inc., Tokyo, Japan).
