# Supplementary material for: Effects of green light-emitting diode irradiation on hepatic differentiation of hepatocyte-like cells generated from human adipose-derived mesenchymal cells
Source: Sci Rep. 2023 Nov 15;13:19954. doi: 10.1038/s41598-023-45967-7 (PMC10651838; doi:10.1038/s41598-023-45967-7)
Supplement: Supplementary file 2 — Supplementary Figure 2. [file 41598_2023_45967_MOESM2_ESM.docx]

***Effects of green light-emitting diode irradiation on hepatic differentiation of hepatocyte-like cells generated from human adipose-derived mesenchymal cells.***

Yuhei Waki MD, Yu Saito MD, PhD, FACS^*^, Shuhai Chen MD, Tetsuya Ikemoto MD, PhD, FACS, Takayuki Noma MD, Hiroki Teraoku MD, PhD, Shinichiro Yamada MD, PhD, FACS, Yuji Morine MD, PhD, FACS, Mitsuo Shimada MD, PhD, FACS

Department of Surgery, Tokushima University, 3-18-15 Kuramoto-cho, Tokushima 770-8503, Japan

**
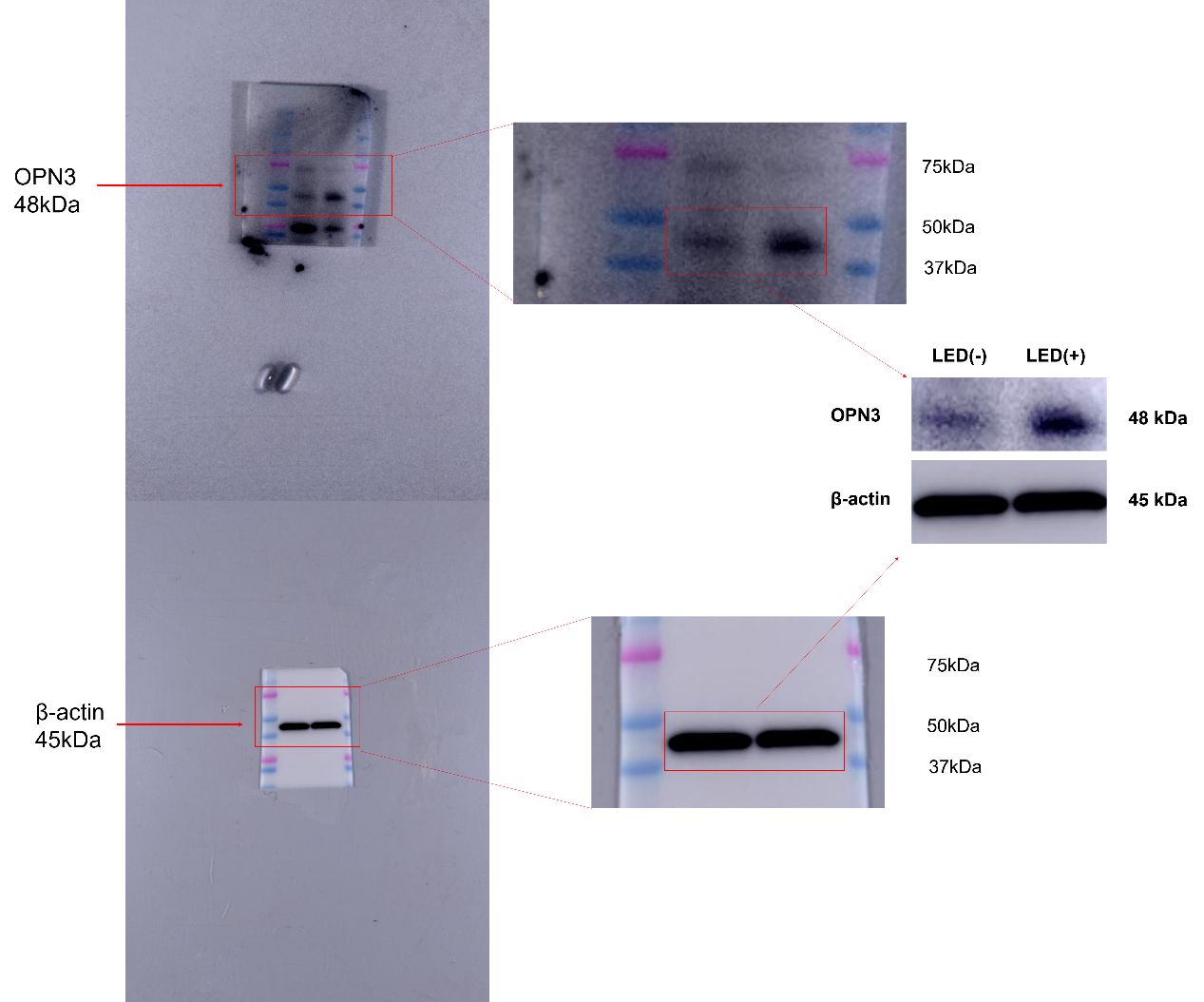
**

**Supplementary Figure 2. Original Western blot blots of Figure 4b**

Western blots were cropped prior to incubation with primary antibody hybridization. The left side figures show original blots with the position of the bands when exposed to Western blot. Since the molecular weights of OPN3 and β-actin are similar, separate blots were used for the same sample with exposure times of 3 minutes for OPN3 and 15 seconds for β-actin. The middle figures show the molecular weight of the marker bands. The bands were extracted separately and combined to form the Figure 4b plot on the right.
