# Supplementary material for: Effects of green light-emitting diode irradiation on hepatic differentiation of hepatocyte-like cells generated from human adipose-derived mesenchymal cells
Source: Sci Rep. 2023 Nov 15;13:19954. doi: 10.1038/s41598-023-45967-7 (PMC10651838; doi:10.1038/s41598-023-45967-7)
Supplement: Supplementary file 3 — Supplementary Figure 3. [file 41598_2023_45967_MOESM3_ESM.docx]

***Effects of green light-emitting diode irradiation on hepatic differentiation of hepatocyte-like cells generated from human adipose-derived mesenchymal cells.***

Yuhei Waki MD, Yu Saito MD, PhD, FACS^*^, Shuhai Chen MD, Tetsuya Ikemoto MD, PhD, FACS, Takayuki Noma MD, Hiroki Teraoku MD, PhD, Shinichiro Yamada MD, PhD, FACS, Yuji Morine MD, PhD, FACS, Mitsuo Shimada MD, PhD, FACS

Department of Surgery, Tokushima University, 3-18-15 Kuramoto-cho, Tokushima 770-8503, Japan

**
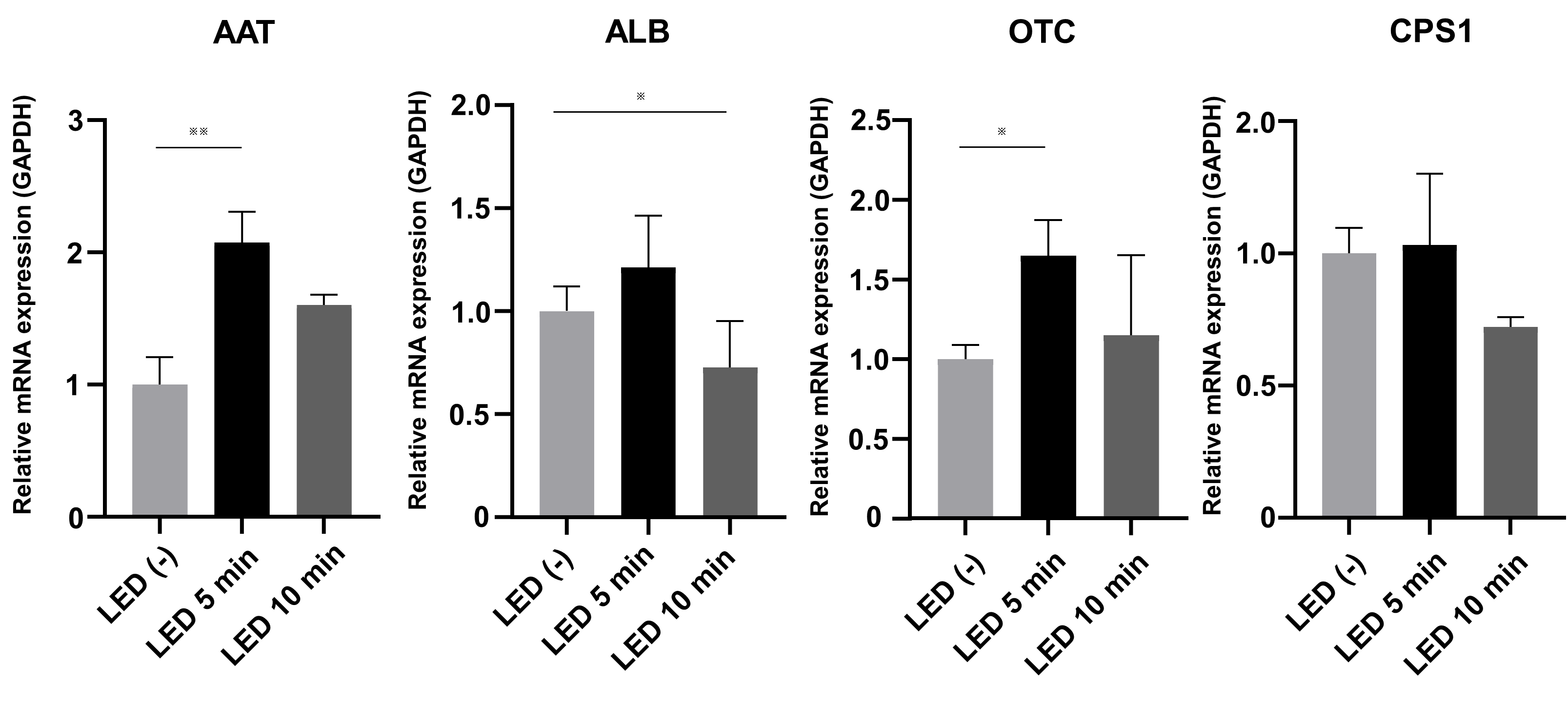
Supplementary Figure 3. Effects of GLED irradiation time for HLCs.**

Gene expression of AAT and OTC was significantly upregulated in GLED-irradiated HLCs for 5 mins compared with non-irradiated HLCs. The data are shown as means ± standard deviation. N.S. not significant.
