# Supplementary material for: Effects of green light-emitting diode irradiation on hepatic differentiation of hepatocyte-like cells generated from human adipose-derived mesenchymal cells
Source: Sci Rep. 2023 Nov 15;13:19954. doi: 10.1038/s41598-023-45967-7 (PMC10651838; doi:10.1038/s41598-023-45967-7)
Supplement: Supplementary file 4 — Supplementary Table 1. [file 41598_2023_45967_MOESM4_ESM.docx]

***Effects of green light-emitting diode irradiation on hepatic differentiation of hepatocyte-like cells generated from human adipose-derived mesenchymal cells.***

Yuhei Waki MD, Yu Saito MD, PhD, FACS^*^, Shuhai Chen MD, Tetsuya Ikemoto MD, PhD, FACS, Takayuki Noma MD, Hiroki Teraoku MD, PhD, Shinichiro Yamada MD, PhD, FACS, Yuji Morine MD, PhD, FACS, Mitsuo Shimada MD, PhD, FACS

Department of Surgery, Tokushima University, 3-18-15 Kuramoto-cho, Tokushima 770-8503, Japan

**Supplementary Table 1. CT values of GAPDH in ADSC, HLC and PHH**

| CT values | ADSC | LED (-) HLC | LED (+) HLC | PHH |
| --- | --- | --- | --- | --- |
| Mean | 20.819 | 20.866 | 21.008 | 20.924 |
| Standard deviation | 0.065 | 0.134 | 0.132 | 0.057 |
| CT, cycle threshold, ADSC, adipocyte derived stem cell, LED, light emitted diode, HLC, hepatocyte like cell, PHH, primary human hepatocyte | | | | |
